# The dynamics of E1A in regulating networks and canonical pathways in quiescent cells

**DOI:** 10.1186/1756-0500-4-160

**Published:** 2011-05-26

**Authors:** Jean-Eudes Dazard, Keman Zhang, Jingfeng Sha, Omar Yasin, Linda Cai, Chien Nguyen, Mrinal Ghosh, Jennifer Bongorno, Marian L Harter

**Affiliations:** 1 Center for Proteomics and Bioinformatics, Case Western Reserve University, Cleveland, Ohio 44106, USA; 2 Department of Biochemistry, School of Medicine, Case Western Reserve University, Cleveland, Ohio 44106, USA; 3 Division of Cell Biology & Physiology, Indian Institute of Chemical Biology, West Bengal, India; 4 The Case Comprehensive Cancer Center, Case Western Reserve University, Cleveland, Ohio 44106, USA

## Abstract

**Background:**

Adenoviruses force quiescent cells to re-enter the cell cycle to replicate their DNA, and for the most part, this is accomplished after they express the E1A protein immediately after infection. In this context, E1A is believed to inactivate cellular proteins (e.g., p130) that are known to be involved in the silencing of E2F-dependent genes that are required for cell cycle entry. However, the potential perturbation of these types of genes by E1A relative to their functions in regulatory networks and canonical pathways remains poorly understood.

**Findings:**

We have used DNA microarrays analyzed with Bayesian ANOVA for microarray (BAM) to assess changes in gene expression after E1A alone was introduced into quiescent cells from a regulated promoter. Approximately 2,401 genes were significantly modulated by E1A, and of these, 385 and 1033 met the criteria for generating networks and functional and canonical pathway analysis respectively, as determined by using Ingenuity Pathway Analysis software. After focusing on the highest-ranking cellular processes and regulatory networks that were responsive to E1A in quiescent cells, we observed that many of the up-regulated genes were associated with DNA replication, the cell cycle and cellular compromise. We also identified a cadre of up regulated genes with no previous connection to E1A; including genes that encode components of global DNA repair systems and DNA damage checkpoints. Among the down-regulated genes, we found that many were involved in cell signalling, cell movement, and cellular proliferation. Remarkably, a subset of these was also associated with p53-independent apoptosis, and the putative suppression of this pathway may be necessary in the viral life cycle until sufficient progeny have been produced.

**Conclusions:**

These studies have identified for the first time a large number of genes that are relevant to E1A's activities in promoting quiescent cells to re-enter the cell cycle in order to create an optimum environment for adenoviral replication.

## Background

Most somatic cells, including adult stem cells, are in a non-dividing or quiescent state (G0), and except for those that have become terminally differentiated or senescent, they can still re-enter the cell cycle when necessary. The molecular pathways that are responsible for maintaining cellular quiescence are largely unknown. However, it is known that these pathways can be influenced by external stimuli such as nutrients or growth factors, and that this in turn allows quiescent cells to grow, progress through G1, and ultimately proliferate.

Human adenoviruses are another factor that can affect the pathways that control cellular quiescence [1]. These DNA viruses, which are a causative agent for various types of human diseases, typically infect non-dividing cells and force them into S phase for replicating DNA. Ultimately, the viruses then use the cellular DNA precursors and the host enzymes to replicate their own DNA [2]. The first viral gene to be transcribed following adenovirus infection is E1A, and it encodes two major proteins of 289 (289R) and 243 amino acids (243R) [2]. The smaller size E1A is principally responsible for transitioning either human or rodent cells out of quiescence, and it can perform this function either alone or in the context of the virus [2-5]. This effect correlates with its ability to target key cellular proteins involved in regulating the cell cycle and chromatin function. Included in this group of proteins are the retinoblastoma family (pRb, p107, and p130), inhibitors of cyclin-dependent kinases, histone acetyltransferases, and other chromatin factors [2,6,7].

The proteins pRb and p130 are especially important since they are both highly involved in regulating the E2F family of transcription factors (E2F1-E2F5). The E2F1-3a factors are activators of transcription and bind exclusively to pRb, whereas p130 interacts specifically with E2F repressors such as E2F4 [8]. Studies have shown that the repressor complex p130-E2F4 associates with a substantial number of E2F-dependent genes in quiescent cells and that it serves to silence these genes by recruiting histone-modifying enzymes such as deacetylases (HDACs) and methyltransferases [9-11] to their respective promoters. The genes that are regulated by this complex include many that are involved in DNA replication, cell cycle control, and metabolism [11]. We have recently found that when expressed in quiescent cells, E1A can reverse the repression of at least two of these genes (e.g., *CDC6 *and *CCNA*) by eliminating p130-E2F4 and HDAC complexes from their promoters, and then by recruiting a histone acetyltransferase to acetylate the surrounding nucleosomal histones [4,5].

There are numerous E2F-dependent genes that are involved in a variety of biological processes [12], and when considering our previous work [4,5], many of these have the potential to be targeted by E1A, leading to de-repression of their expression in quiescent cells. E1A thus gives us an opportunity to identify, on a global scale, the gene regulatory networks that are required by the virus for its propagation in these cells. We have therefore used DNA microarray analyses with well-established statistical approaches on hybridized data from quiescent cells with or without the expression of E1A in order to address this important issue.

## Results and Discussion

### General Strategy

Our previous studies have described a "Tet-on" inducible mouse cell line (Balb/c 3T3) that expresses the adenovirus E1A-243R protein in a regulated manner [4,5] An important feature of this cell line is that it can be brought to a state of quiescence by either contact inhibition or mitogen deprivation. Published data show that in this state, at least 95% of the cells are no longer in S phase, as measured by the absence of BrdU incorporation [4,5]. Once in this state, however, these cells can be made to transition into S phase by the induction of E1A following the addition of doxycycline (Dox), a tetracycline analog. In such an experiment, we typically find that 35-40% of the cells are incorporating BrdU after being treated with Dox for 12 hr, and > 98% of them express E1A [4]. Moreover, a microinjected E1A when compared to serum stimulation shortens the transition from G0 to S phase in quiescent cells with DNA synthesis beginning as early as 7 hr after its expression [13]. Because our E1A-inducible cell line gives us the opportunity to identify E1A target genes in an unbiased and exclusive manner, we decided to use it in combination with DNA microarray analysis in order to identify, on a global scale E1A-mediated differentially expressed genes in quiescent cells.

### Statistical Approach

Six independent cultures (*n *= 6) were used for cRNA labelling for the transcriptional profiling. The categorical factor under study consisted of a single 2-level *Group Factor *(GF): the control group, denoted 'Q' (quiescent cells without Dox, and therefore no E1A expression), versus the stimulated group, denoted 'S' (stimulated cells with Dox, and therefore with E1A expression). In this design, cell cultures were randomly assigned to treatments with three independent biological replicates per group. Therefore, this is an arrangement of treatments laid out on a balanced Complete Randomized Design (CRD) with no repeated measurements. In addition, no technical replicates were performed, no pooling was done, and there was no common reference sample.

One of the goals in high dimensional data mining is to identify which of the variables (such as mRNA and EST probes - sometimes abbreviated as genes) show evidence of differential expression between experimental conditions, while dealing with high dimensional contextual problems. To detect differential expression between experimental conditions when the number of variables greatly exceeds the number of observations or samples (*p *>>*n *paradigm), conventional regression techniques literally fall apart or are inappropriate at best. A standard approach in modelling high dimensional data is to fit the same statistical model individually to each outcome variable and test for the contrast or effect of interest using the hypothesis testing framework. Among the drawbacks of this univariate approach are the correlation structure (i.e. dependency) between the variables, which is totally ignored, while the risk of excessive conservativeness and of over fitting can be severely inflated [14].

In this study, we took advantage of the fact that the problem of differential expression can be cast into a variable selection problem in a regression setting. Recently, Bayesian model selection methods were proven the most reasonable approach to detect differentially expressed genes in high dimensional settings [15-17]. We employed one of these methods called Bayesian ANOVA (BAM). In effect, BAM is a model selection technique that relies on the so-called 'spike and slab' Bayesian hierarchical model used in parameter estimation. It is a special type of inferential regularization (i.e. borrowing information across genes), which builds a parsimonious model by selectively shrinking to zero only those (model) coefficients of genes that truly do not enter in the model [15]. This is an ideal property guaranteeing which genes will enter into the model and which will not, and allowing for optimal balancing of the number of false detections against false non-detections (i.e. false positive and false negative rates, or total gene misclassification errors), thereby leading to a more accurate and parsimonious model of truly differentially expressed genes [16,17].

Moreover, this technique is far superior to conventional one-at-a-time (univariate) hypothesis testing procedures, followed by multiple testing corrections that attempt to control only False Detection Rates (*FDR*) [16,17]. This is because controlling *FDR *tends to identify obviously varying genes but misses more subtle changes. This method also eliminates the problem of specifying arbitrarily False Discovery Rate (*FDR*) cutoff values, and the drawback of excessive conservativeness [14]. In addition, BAM does not ignore the problem of dependency between the variables, which is frequently present within large datasets, and in biological data. Therefore, by using the BAM model, we were able to define a subset of differentially expressed genes of high statistical significance in response to E1A's expression in quiescent cells (see below).

### Microarray Analysis Identifies Genes that are modulated by E1A in Quiescent Cells

To reveal differentially regulated genes in quiescent cells after E1A expression, we hybridized cRNA prepared from quiescent cells treated with or without Dox for 8 hr onto Agilent Whole Mouse Genome arrays, which allows for the interrogation of ~25,000 genes. This time point is approximately 1 hr after the start of E1A-mediated DNA synthesis in these cells, and therefore considers only genes that changed significantly in expression after cells transitioned from G0 into S phase.

**Figure 1:**
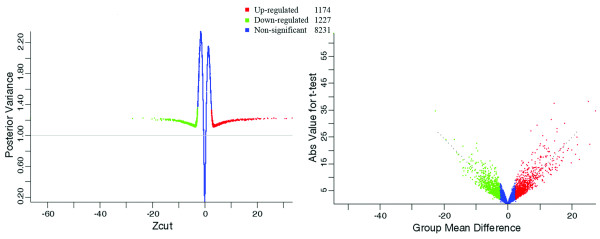
**Data plots for assessing differentially expressed genes in quiescent Balb/c 3T3 cells after E1A induction**. The left panel represents a Shrinkage Plot where every point represents a gene. Red dots and green dots indicate genes that were up regulated and down regulated, respectively, while the blue dots represent those genes that were non-regulated. The horizontal axis shows BAM *Zcut *values of expression changes while the vertical axis shows the corresponding *Posterior Variances*. The right panel is a diagnostic volcano plot of the comparison between quiescent Balb/c 3T3 cells with or without the expression of E1A. In this plot, each individual gene is arranged by statistical significance, and the most significant of these are those that have the largest estimated magnitude of change (i.e. *Group Mean Difference*) and the largest absolute value *t*-test (distributed in the top right or left of the plots). The genes that were significantly up regulated are highlighted in red, and those that were significantly down regulated are highlighted in green.

**Figure 2:**
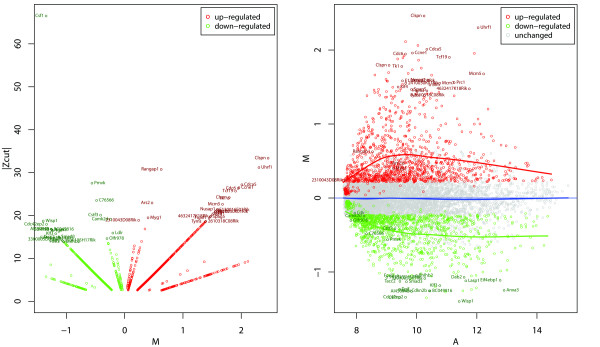
**Mapping of differentially expressed genes onto Volcano and MA plots**. Genes significantly regulated by E1A and found to be up-regulated or down-regulated by BAM analysis are highlighted in red or green, respectively, while the non-regulated genes are shown in grey. The top 50 regulated genes (up or down) from Table 1 are named along with a few un-annotated genes called Rik identifiers. Left panel: volcano plot of absolute BAM Zcut values plotted versus log-fold-change-ratios on a log-equivalent transformed scale, denoted M = glog(S/Q), where (S) denotes cells with E1A and (Q) without E1A. Right panel: MA plot. Vertical and horizontal axis are the log-fold-change-ratios on a log-equivalent transformed scale, denoted M = glog(S/Q), and the log-geometric-means on a log-equivalent transformed scale, denoted A = ½*glog(S*Q) respectively. Solid lines in the MA plot represent the LOESS smoothing curves of the differentially expressed genes (red and green) and non-regulated genes (blue).

To reduce the dimensionality of the problem (i.e., the number of potential probes at play on the microarray), we initially applied a pre-filtering step as described in Materials and Methods section, thereby reducing the number of probes to 10,632. After carrying out our Bayesian ANOVA on those 'Present' genes, we were able to select 2,401 genes on the arrays whose expression showed a significant change after quiescent cells had been treated with Dox [Figure 1 and Additional file 1: Supplemental Table S1]. Eight of these genes were unnamed, and 1,174 of the genes were up regulated, while 1,227 of them were down regulated. The total number of E1A-regulated genes is displayed in a Volcano and MA plot [Figure 2] as well as in a normalized quantile-quantile plot [Additional file 2: Supplemental Figure S1]. These plots importantly show (i) that only a few of these genes would have been discovered if only a conventional fold change analysis had been used [Figure 2 and Additional file 2: Supplemental Figure S1], and (ii) that conventional statistical approaches such as ANOVA or *t*-test would be inappropriate because of the violation of the normality assumption [Additional file 2: Supplemental Figure S1]. The top 50 genes that were up regulated or down regulated by this analysis are listed in Table 1. This analysis of E1A's activity in promoting quiescent cells into S phase has therefore allowed us to uncover, for the first time, significant factors to induce this event.

**Table 1:** List of top twenty up- and down-regulated genes found differentially expressed by BAM analysis in quiescent cells after E1A expression (Dox-treatment).

Accession	Symbol	Definition	*Zcut*	glog(*FC*)
**Down-Regulated Genes**				
NM_007778.1	Csf1	Mus musculus colony stimulating factor 1 (macrophage)	-66.5094	-1.348
NM_026784.1	Pmvk	Mus musculus phosphomevalonate kinase	-27.5436	-0.562
NM_178879.2	C76566	Mus musculus expressed sequence C76566	-23.5382	-0.482
NM_145529.1	Cstf3	Mus musculus cleavage stimulation factor, 3 pre-RNA, subunit 3	-20.1076	-0.413
NM_023813.2	Camk2d	Mus musculus calcium/calmodulin-dependent protein kinase II, delta	-19.1488	-0.242
NM_018865.1	Wisp1	Mus musculus WNT1 inducible signaling pathway protein 1	-18.7272	-1.396
NM_026772.1	Cdc42ep2	Mus musculus CDC42 effector protein (Rho GTPase binding) 2	-17.9546	-1.340
NM_001033476.1	AI450948	Mus musculus expressed sequence AI450948	-16.851	-1.261
NM_198612.1	BC049816	Mus musculus cDNA sequence BC049816	-16.7728	-1.255
NM_010212	Fhl2	Mus musculus four and a half LIM domains 2	-16.7683	-1.254
NM_007670.2	Cdkn2b	Mus musculus cyclin-dependent kinase inhibitor 2B (p15)	-16.7461	-1.253
NM_013470.1	Anxa3	Mus musculus annexin A3	-16.6472	-1.246
NM_009372.2	Tgif	Mus musculus TG interacting factor	-16.4772	-1.234
NM_010700.1	Ldlr	Mus musculus low density lipoprotein receptor	-15.9324	-0.203
NM_008453.2	Klf3	Mus musculus Kruppel-like factor 3 (basic)	-15.7751	-1.183
NM_016769	Smad3	Mus musculus SMAD family member 3	-15.0449	-1.130
NM_206856.1	Tacc2	Mus musculus transforming, acidic coiled-coil containing protein 2	-14.9903	-1.126
NM_010688.2	Lasp1	Mus musculus LIM and SH3 protein 1	-14.8866	-1.119
NM_007918.2	Eif4ebp1	Mus musculus eukaryotic translation initiation factor 4E binding protein 1	-14.7742	-1.111
NM_147105.1	Olfr978	Mus musculus olfactory receptor 978	-14.6434	-0.304
XM_196166	3300005D01Rik	Mus musculus RIKEN cDNA 3300005D01 gene	-14.4319	-1.086
NM_023118.1	Dab2	Mus musculus disabled homolog 2 (Drosophila)	-14.2169	-1.071

**Up-Regulated Genes**				

NM_175554.3	Clspn	Mus musculus claspin homolog	33.3736	1.378
NM_010931.2	Uhrf1	Mus musculus ubiquitin-like, containing PHD and RING finger domains, 1	31.1992	1.382
NM_011241	Rangap1	Mus musculus RAN GTPase activating protein 1	30.7663	1.384
NM_026410.1	Cdca5	Mus musculus cell division cycle associated 5	27.1852	1.387
NM_007633.1	Ccne1	Mus musculus cyclin E1	26.512	1.390
NM_011799.1	Cdc6	Mus musculus cell division cycle 6 homolog (S. cerevisiae)	26.3215	0.239
NM_025674.1	Tcf19	Mus musculus transcription factor 19	25.7018	1.449
NM_175554.3	Clspn	Mus musculus claspin homolog	24.2127	0.400
NM_009387	Tk1	Mus musculus thymidine kinase 1	23.9996	1.468
NM_031405.1	Ars2	Mus musculus arsenate resistance protein 2	22.9415	1.477
NM_008566.1	Mcm5	Mus musculus minichromosome maintenance deficient 5, cell division cycle 46	22.6281	1.502
NM_133851.1	Nusap1	Mus musculus nucleolar and spindle associated protein 1	21.4676	1.529
NM_001039556.1	E130016E03Rik	Mus musculus RIKEN cDNA E130016E03 gene	21.3155	1.552
NM_145150.1	Prc1	Mus musculus protein regulator of cytokinesis 1	20.9803	1.553
NM_008568.1	Mcm7	Mus musculus minichromosome maintenance deficient 7 (S. cerevisiae)	20.8883	1.560
NM_026282.2	2410030K01Rik	Mus musculus RIKEN cDNA 2410030K01 gene	20.879	1.584
NM_007550	Blm	Mus musculus Bloom syndrome homolog (human)	20.5591	1.594
NM_008446.1	Kif4	Mus musculus kinesin family member 4	20.1831	1.679
NM_026640.1	4632417K18Rik		19.8413	0.470
NM_017407.1	Spag5	Mus musculus sperm associated antigen 5	19.7147	1.779
NM_021713.1	Myg1	Mus musculus melanocyte prolifeating gene 1	19.4864	1.794
NM_021891.2	Fignl1	Mus musculus fidgetin-like 1	19.4541	1.903
XM_130428.2	2310043D08Rik		18.9002	1.948
XM_149213.1	2610318C08Rik	Mus musculus RIKEN cDNA 2610318C08 gene	18.6426	1.961
NM_021288.2	Tyms	Mus musculus thymidylate synthase	18.5966	2.010
NM_177733.2	E2f2	Mus musculus E2F transcription factor 2	18.5614	0.627
NM_007891.1	E2f1	Mus musculus E2F transcription factor 1	18.5401	2.303
NM_011284.2	Rpa2	Mus musculus replication protein A2	18.4827	2.461

Transcripts are ranked by statistical significance (*Zcut *values) from top to bottom. Direction of change is indicated by the *Zcut *sign. Generalized-log-fold change values (glog(*FC*)) represent the corresponding *magnitude *of regulation of the selected genes.

We next employed independent qRT-PCR assays to validate the array results for genes that displayed high or low fold change values, and which are known to have a direct or indirect role in DNA replication or cell cycle progression [Additional file 3: Supplemental Figure S2]. In the genes that were studied, and in the statistical methods that were employed, both of these technological platforms yielded changes that were proportionally conserved in their expression, indicating the reproducibility of change and the reliability of detection by microarray analysis.

These findings, along with our previous results [4,5], confirm that E1A alone has the capacity to affect ~2,400 genes in quiescent cells. As shown below, some of these genes are strictly related to maintaining cellular quiescence, while others are more important for helping cells to transition out of quiescence. It is worth noting that the number of genes (~6,400) whose promoters were reported to be bound by E1A in quiescent human cells, as determined by ChIP-on-chip analysis [18] is considerably larger than the number of genes found to be affected by E1A in our study. The basis for this difference is unknown, although the ChIP-on-chip analysis was performed on cells that had been infected with adenovirus for a period of 6 hr. In addition, the binding of E1A to promoters does not directly and immediately have to affect the expression of the respective genes. Another gene expression-profiling experiment using adenovirus infected quiescent human cells identified ~2,100 differentially expressed genes [1]. Interestingly, the earliest change in the concentrations of cellular RNA found in these cells was at 18 hr post-infection, at least 6 hr beyond the time when E1A was expressed [1]. Although E1A is likely involved in deregulating many of the cellular genes reported in the aforementioned studies, there is still the possibility of other viral proteins being contributors to this deregulation as well

### E1A Regulates a Variety of Biological Processes to Induce Cells out of Quiescence

In order to identify the biological processes, canonical pathways and molecular networks that are potentially regulated by E1A for transitioning cells out of quiescence, we up-loaded the BAM Zcut values of the E1A-modulated genes into the Ingenuity Pathway (IPA) software. We applied specific filters (see Materials and Methods) for limiting our experimental observations to information that would be most relevant to the reversibility of quiescent cells, and which left 385 up/down-regulated molecules/genes that were eligible for generating networks, and 1033 molecules/genes, which were eligible for functional and canonical pathway analysis. Of interest is that regardless of whether the IPA filter for mouse or human was used, the results from the respective analyses were strikingly similar.

**Figure 3:**
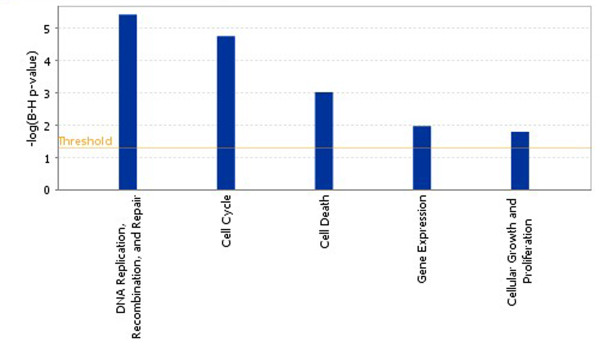
**Molecular and cellular functions most significantly regulated by E1A expression in quiescent cells**. The Benjamini-Hochberg (BH) method was used to adjust the right-tailed Fisher's Exact *t*-test *p*-values, which measure how significant each pathway is. Functions having the highest *p*-values are shown. For a complete listing of the genes used in this analysis, see Supplemental Table S1 [Additional file 1: Supplemental Table S1].

After performing an IPA core analysis, we found that the E1A-243R was able to modulate sets of genes that are known to function in several key cellular processes, and which were either upregulated or downregulated in their expression (Figure 3). In particular, genes involved in transitioning cells out of quiescence (e.g., *CDK2, E2F1, E2F2, RBL1, RB1, ID2, SKP2, BRAC2, TP53, CCNE1*, *CDC25C*, and *ATM*) and for making chromatin competent for DNA replication (e.g., *CDC6, CDT1*, the MCM replicative helicase complex (*Mcm2-Mcm7*, *ORC1L, and ORC6L*) were significantly up-regulated after E1A was expressed in quiescent cells [Additional file 1: Supplemental Table S1]. We also identified several other genes which were significantly up regulated and are known to be required for the metabolism and synthesis of DNA [19]. These included *POLD1, POLA2, FEN1, KIN, PRIM1, PRIM2, HELB, NASP*, and *LIG1*. Notably, the promoter regions of most of these genes are known to be occupied by the E2F4 repressor complex in quiescent cells [20]. Given that, E1A alone can dissociate this transcriptional repressor from an inactive endogenous CDC6 promoter in quiescent cells [4,5], this new data suggests that E1A may also have the ability to directly affect the promoters of other genes that are subject to E2F4 repression in these cells. In effect, this may be one way in which E1A contributes to the growth of viruses in the infected cells.

Although there is evidence to suggest that quiescent mouse cells lack global genomic DNA repair, they do exhibit proficient transcription-coupled DNA repair [21,22]. Interestingly, the IPA analysis identified clusters of up regulated genes that are involved in different DNA repair pathways, as well as in checkpoint controls, and the DNA damage response (DDR) (Table 2). As noted in the Table, a majority of the genes can bind E2F4 [12], and at least one-third of them have been found to be associated with this factor in quiescent cells [20], and therefore have the potential of being up regulated by E1A.

**Table 2:** Identification of up regulated genes identified in E1A expressing quiescent cells and required for DNA repair, checkpoint controls, and the DNA damage response.

*Accession*	*Gene Symbol*	*Description*	*E2F4 binding*^1,2^	*Zcut*	*glog(FC)*
		***DNA repair processes: homologous recombination, mismatch/excision repair, and non-homologous end-joining***			
NM_007499.1	ATM	Ataxia telangiectasia mutated	**+**	7.498	0.585
NM_007550	BLM	Bloom syndrome, RecQ helicase-like	**+**	20.559	1.529
NM_009764.2	BRCA1	Breast cancer 1, early onset	-	4.757	0.381
NM_009765.1	BRCA2	Breast cancer 2, early onset	-	12.234	0.927
NM_007691.2 NM_009863.1	CHEK1 CDC7	CHK1 checkpoint homolog (S. Pombe) Cell division cycle 7 homolog (S. cerevisiae)	**+ +**	3.202 17.694	0.261 1.321
NM_028119.2	DDB2	Damage-specific DNA binding protein 2, 48 kDa	**+**	9.204	0.709
NM_177752.2	EME1	Essential meiotic endonuclease 1 homolog 1 (S. pombe)	-	2.820	0.232
NM_001033244.1	FANCD2	Fanconi anemia, complementation group D2	**+**	9.508	0.730
NM_007999.2	FEN1	Flap structure-specific endonucleae 1	**+**	8.577	1.957
NM_010436.2	H2AFX	H2A histone family, member X	**+**	4.634	1.095
NM_010715.1	LIG1	Ligase 1, DNA, ATP-dependent	**+**	18.060	1.348
NM_026810.1	MLH1	MutL homolog 1, colon cancer, nonpolyposis type 2 (E. coli)	**+**	2.794	0.230
NM_018736.2	MRE11A	MRE11 meiotic recombination 11 homolog A (S. cerevisiae)	-	5.432	0.432
NM_008628.1	MSH2	MutS homolog 2, colon cancer, nonpolyposis type 1 (E. coli)	**+**	3.107	0.254
NM_010830.1	MSH6	MutS homolog 6 (E.coli)	-	13.241	1.000
NM_013752	NBN	Nibrin	-	8.326	0.645
NM_009632.2	PARP2	Poly (ADP-ribose) polymerase 2	**+**	7.656	0.596
NM_011045.1	PCNA	Proliferating cell nuclear antigen	**+**	10.999	0.838
NM_008886.1	PMS2	PMS2 postmeiotic segregation increased 2 (S. cerevisiae)	-	8.409	0.651
NM_011131.2	POLD1	Polymerase (DNA directed) delta 1, catalytic subunit 125 kDa	**+**	7.744	0.603
NM_030715.2 NM_008949.2	POLH PSMC3IP	Polymerase (DNA directed), eta PSMC3 interacting protein	**- +**	3.448 17.496	0.825 1.307
NM_013917.1 NM_021385.1	PTTG1 RAD18	Pituitary tumor-transforming 1 RAD18 homolog (S. cerevisiae)	**+ +**	7.190 7.629	0.562 0.594
NM_009012.1	RAD50	RAD50 homolog (S. cerevisiae)	**+**	4.064	0.328
NM_011234.2	RAD51	RAD51 homolog (RecA homolog, E. coli) (S. cerevisiae)	**+**	12.514	0.947
NM_009013.1	RAD51AP1	RAD51 associated protein 1	**+**	9.638	0.740
NM_009015.2	RAD54L	RAD54-like (S. cerevisiae)	**+**	9.320	2.115
NM_026653.1	RPA1	Replication protein A1, 70 kDa	-	9.177	0.707
NM_011237.1	RAD9A	RAD9A homolog A	-	4.501	0.361
NM_023042.1	RECQL	RecQ protein-like (DNA helicase Q1-like)	**+**	5.724	0.125
NM_021419.1	RNF8	Ringfinger protein 8	**+**	2.663	0.222
NM_011677.1	UNG	Uracil-DNA glycosylase	**+**	4.901	0.475
NM_133786.3 NM_153808.1	SMC4L1 SMC5L1	Structural maintenance of chromosomes four-like 1 Structural maintenance of chromosomes five-like 1	**- -**	4.290 10.047	0.345 0.769
XM_127444.3 NM_010247.1	TRIP13 XRCC6	Thyroid hormone receptor interactor 13 X-ray repair complementing defective repair in Chinese hamster	**+ -**	3.630 3.039	0.294 0.732
					

***Accession***	***Gene Symbol***	***Description***	***E2F4 binding^1,2^***	***Zcut***	***glog(FC)***

		***DNA damage response***			
NM_007499.1	ATM	Ataxia telangiectasia mutated	**+**	7.498	0.585
NM_009764.2	BRCA1	Breast cancer 1, early onset	-	4.757	0.381
NM_009765.1	BRCA2	Breast cancer 2, early onset	-	12.234	0.927
NM_007691.2	CHEK1	CHK1 checkpoint homolog (S. Pombe)	**+**	3.202	0.261
NM_001033244.1	FANCD2	Fanconi anemia, complementation group D2	**+**	9.508	0.730
NM_008316.2	HUS1	HUS1 checkpoint homolog (S. pombe)	-	4.438	0.357
NM_010774.1	MBD4	Methyl-CpG binding domain protein 4	-	8.816	0.681
NM_008583.1	MEN1	Multiple endocrine neoplasia 1	**?**	4.995	0.399
NM_026810.1	MLH1	MutL homolog 1, colon cancer, nonpolyposis type 2 (E. coli)	**+**	2.794	0.230
NM_008628.1	MSH2	MutS homolog 2, colon cancer, nonpolyposis type 1 (E. coli)	**+**	3.107	0.254
NM_010830.1	MSH6	Muts homolog 6 (E. coli)	-	13.241	1.000
NM_008884.2	PML	Promyelocytic leukemia	-	3.155	0.257
NM_011237.1	RAD9A	RAD9 homolog A (S. pombe)	-	4.501	0.361
NM_011234.2	RAD51	RAD51 homolog (RecA homolog, E. coli) (S. cerevisiae)	**+**	12.514	0.947
NM_009015.2	RAD54L	RAD54-like (S. cerevisiae)	**+**	9.320	2.115
NM_026653.1	RPA1	Replicaton protein A1, 70 kDa	**+**	9.177	0.707
NM_011623.1	TOP2A	Topoisomerase (DNA) 11 alpha 170 kDa	**+**	8.224	1.880
NM_011640.1	TP53	Tumor protein p53	-	6.333	0.499
NM_010247.1	XRCC6	X-ray repair complementing defective repair in Chinese hamster	-	3.039	0.732

***Accession***	***Gene Symbol***	***Description***	***E2F4 binding^1,2^***	***Zcut***	***glog(FC)***

		***DNA replication checkpoints and checkpoint control***			
NM_007499.1	ATM	Ataxia telangiectasia mutated	**+**	7.498	0.585
NM_026014.3	CDT1	Chromatin licensing and DNA replication factor 1	-	4.137	0.983
NM_007691.2 NM_008316.2	CHEK1 HUS1	CHK1 checkpoint homolog (S. Pombe) HUS1, checkpoint homolog (S. pombe)	**+ +**	3.202 4.438	0.261 0.357
NM_008583.1	MEN1	Multiple endocrine neoplasia 1	-	4.995	0.399
NM_008628.1	MSH2	MutS homolog 2, colon cancer, nonpolyposis type 1 (E. coli)	**+**	3.107	0.254
NM_134092.2	MTBP	Mdm2, transformed 3T3 cell double minute 2	**+**	9.084	0.700
NM_011237.1	RAD9A	RAD9 homolog A (S. pombe)	**+**	4.501	0.361
NM_011640.1	TP53	Tumor protein p53	-	6.333	0.499

^1,2 ^Data for the binding of E2F4 was obtained from Ren et al. 2002 (superscript 1) and Xu et al. 2007 (superscript 2). Generalized-log-fold change values (glog(*FC*)) represent the corresponding *magnitude *of regulation of the selected genes.

**Figure 4:**
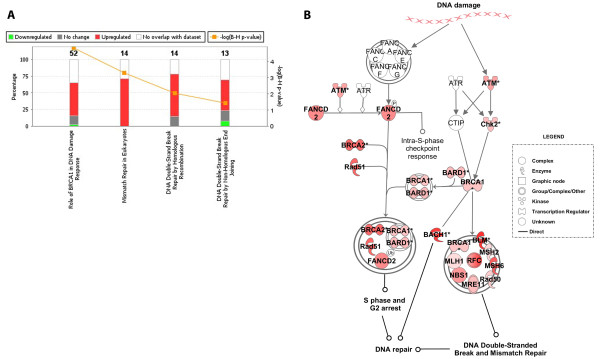
**Canonical pathways most significantly regulated by E1A expression in quiescent cells**. A: The stacked bar chart displays for each canonical pathway the number of genes that were found significantly up-regulated (red), and down-regulated (green) by Bayesian model selection. The molecules/genes in a given pathway that were not found in our list of significantly regulated genes are termed unchanged (grey) or not overlapping with our dataset (white). The numerical value at the top of each bar represents the total number of genes/molecules in the canonical pathway. The Benjamini-Hochberg (BH) method was used to adjust the right-tailed Fisher's Exact *t*-test *p*-values, which measure how significant each pathway is. B: The intensity of the node color (red) indicates the expression level or degree of up regulation, and those genes either in grey or without color were not found in our list of significantly regulated genes.

It is interesting to note that all of the DNA repair genes that are up regulated in response to E1A expression in quiescent cells fall into three categories, mismatch and excision repair (e.g., *MSH2*, *MSH6, MLH1*, *CDC7*, and *PMS2*), and DNA double-strand break repair by homologous recombination (e.g., *ATM, BRCA1, BRCA2, MRE11*, and *LIG1*) or non-homologous end-joining (e.g., *ATM, NBS1, MRE11*, and *XRCC1and 6*). This is underscored and graphically illustrated by additional IPA analyses (Figure 4A and Figure 4B), which identified each of these DNA repair systems (*p *values 7.76E-07, 1.14E-04, and 1.83-02, respectively) as significant categories within *Canonical Pathways*. The necessity of these DNA repair systems during E1A-mediated transition out of quiescence and into S phase, either alone or in the context of virus is unclear. However, it is conceivable that the rapid and abnormal progression into S phase induced by E1A could lead to DNA damage (single-stranded or double-stranded), possibly generated by an instability in the replication forks (e.g., stopping or pausing because of the depletion of the nucleotide pool) as they move away from their respective origins [19]. Alternatively, the restoration of global DNA repair systems following the E1A-mediated re-entry of quiescent cells into the cell cycle could be a natural phenomenon so that the proliferating cells can cope with various kinds of DNA damage, if need be.

Finally, additional analysis by the IPA software revealed a set of genes that were significantly down regulated in the E1A expressing cells [Additional file 1: Supplemental Table S1]. Genes of interest that were affected in the functional category of Cellular Growth and Proliferation included the cyclin dependent kinases inhibitors CDKN2A (p16) and CDKN2B (p15) as well as *PTK2*, *SOCS, TOB1*, and *CAV1*, which has been suggested to play an important role in maintaining cells in a quiescent state [23].

### E1A Regulates Specific Cellular Networks and Canonical Pathways in Quiescent Cells

**Figure 5:**
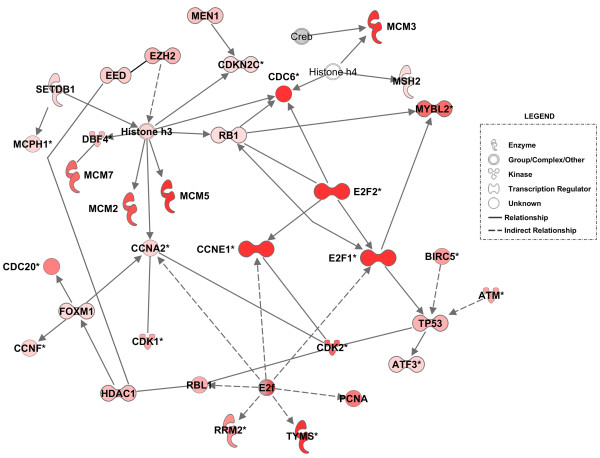
**A network of up-regulated genes in quiescent cells after E1A expression**. The intensity of the node color (red) indicates the expression level or degree of up-regulation. The gene represented in grey (*CREB) *and the ones without color were not found in our list of significantly regulated genes. Both direct (solid line) and indirect (broken line) relationships between the genes are indicated. Cellular functions most significant to the genes in this network included cell cycle re-entry and DNA replication or repair.

To determine whether any of the differentially expressed genes in the E1A expressing cells are interacting with one another, we used the IPA software to generate a listing of gene regulatory networks. Our first analysis was done on the up-regulated genes, and this computation produced one top network that was essentially associated with DNA replication, DNA repair, and the re-entry of cells into the cell cycle. As illustrated in Figure 5, many of the up-regulated genes in this system are involved in DNA replication (*p-value *9.36E-15). For example, genes encoding the activating E2F transcriptional factors (*E2F1 *and *E2F2*), as well as some of the more important components of DNA replication (e.g., *CDC6, MCMs, CCNE1, PCNA*, and *CDK2*) were found to have positions in this network. Genes included in the categories of DNA repair and the response to DNA damage were *MCM7, MEN1, MSH2, TP53, ATM, PCNA TYMS, RRM2*, and *MCPH1*. It should be noted that *MCM7*, in addition to its role in the replication of DNA, might also be involved in the transmission of DNA damage signals [24]. Clearly, this regulatory network not only highlights the interactions between genes that are active in DNA replication but also those that function in DNA repair and DDR. This is consistent with the canonical pathway displayed in Figure 4B. Finally, this network includes *HDAC1*, *EED*, and *EZH2*, which are E2F-dependent genes that participate in the methylation of lysine 27 on histone H3 (H3K27), a marking that is largely associated with the repression of genes [25]. Indeed, a gain in the di- or tri-methylation of H3K27 at specific sites on chromatin might account for at least some of the genes that were down regulated in quiescent cells after E1A expression (Table 1).

**Figure 6:**
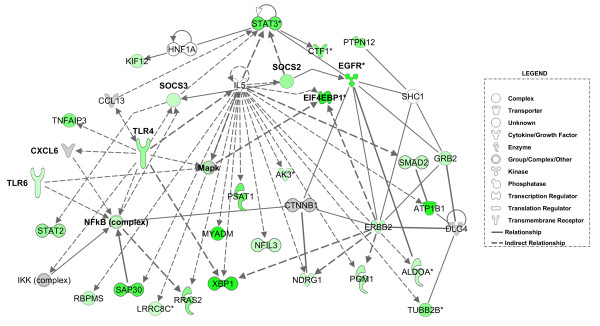
**A network of down-regulated genes in quiescent cells after E1A expression**. The intensity of the node color (green) indicates the expression level or degree of down-regulation. The genes displayed either in grey or without color were not found in our list of significantly regulated genes. *TLR4*, *NFkB *(complex) and *ERBB2 *are important to the formation of this network.

Coordinated interactions between the down-regulated genes in the E1A-modulated dataset were also revealed by using the IPA software. Two of the regulatory networks that were identified by this analysis are presented in Figure 6. Not surprisingly, the genes comprising the first network were linked to biological processes such as cell-to-cell signalling (*p*-value of 6.34E-13), cellular development (*p*-value of 2.57E-12), and cellular growth and proliferation (*p-value *of 6.43E-10). The transcriptional and translational co-repressors *SAP30 *and *EIF4EBP1 *play an important role in this network as does the *SOCS2*, *SOCS3 *and *EGFR*, which are known to increase the differentiation of cells. Of particular interest is that this network also contained the Toll-like receptor proteins *TLR4 *and *TLR6*, which were interconnected with the two signalling pathways (Figure 6). Both of these proteins play a fundamental role in pathogen recognition and the activation of innate immunity [26]. Therefore, their E1A-mediated repression in quiescent cells is provocative since this may represent an additional way of neutralizing the host's antiviral defence. Indeed, it is well established that adenoviruses encode other proteins that function to inhibit host immune responses, for example, killing by CD8^+ ^cytotoxic T lymphocytes [27].

**Figure 7:**
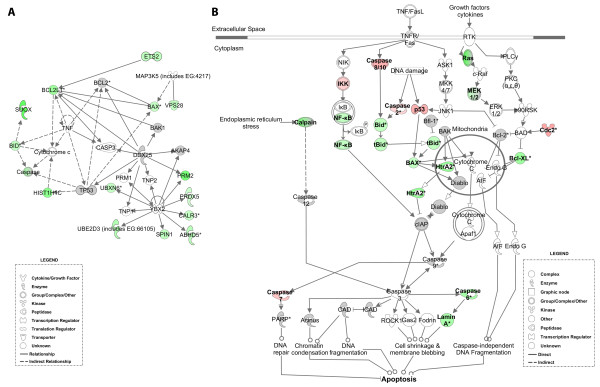
**A network and a canonical pathway of genes affected by E1A in quiescent cells**. A: A network of down-regulated genes identified by BAM. The intensity of the node color (green) indicates the expression level or the degree of down regulation. The genes displayed either in grey (e.g. TP53) or without color were not found in our list of significantly regulated genes. The biological function that was most significant to the genes in this network was cell death. B: Canonical pathway of apoptosis using both up- and down-regulated genes identified by BAM.

The second network displayed in Figure 7A contains down-regulated genes that are primarily involved in apoptosis (*p*-value of 1.02E-17) and developmental processes (*p*-value of 5.46E-16). This network includes a number of pro-apoptotic genes (e.g., *BAX *and *BID*) whose mRNAs decreased in response to E1A expression. The change in expression of these genes and their interactions in apoptotic pathways were also made clear after the down-regulated genes in the database were subjected to canonical pathway analysis. Among the pathways that were revealed in this study, apoptotic signalling (*p*-value of 9.81E-9), was one of the most significant to be perturbed by E1A expression. This suggests that E1A may be blocking p53-independent apoptosis when it is expressed in quiescent cells (Figure 7B). In effect, E1A may be temporarily suppressing the p53-independent apoptosis that has been shown to be a consequence of adenovirus infection, and which is apparently mediated by the larger size E1A protein (289R) and the adenovirus product E4orf4 [28]. Notably, it has been suggested that apoptosis may be promoted by adenovirus to kill cells at the very end of its replicative cycle [28]. This may explain why the virus has developed additional mechanisms, for example the encoding of the E1B-55K and -19K proteins, for controlling apoptosis during the infection.

## Conclusions

One of the most important conclusions to be drawn from the study is the ability of the small size E1A protein of 243R to perturb a rather large number of cellular processes in quiescent cells. This is most likely due to its ability to re-organize chromatin structure [4,5], an effect which in turn leads to the reprogramming of ~ 2400 cellular genes. According to IPA analysis, 271 of these differentially expressed genes are significantly associated with *DNA replication*, *DNA repair*, the *cell cycle *and *cell death*, all of which are necessary for efficient viral replication. This analysis also revealed perturbation in other cellular processes, including *cell-to-cell signalling, cellular development *and *cellular growth*, which may be required to provide an optimal environment for the replication of the virus. Finally, our studies suggest a role for E1A in altering metabolic homeostasis in quiescent cells [Additional file 4: Supplemental Figure S3]. Therefore, a more complete understanding of whether these metabolic differences are necessary for viral growth will be an important goal for future studies.

## Methods

### Cell Culture

The Tet-on inducible Balb/c 3T3 cell line expressing a wild-type 243 amino-acid E1A protein has been previously described [5,29]. This particular clone, which is maintained in DMEM supplemented with antibiotics, L-glutamine, blasticidin/zeocin, and 10% fetal bovine serum (Tet system approved; Clontech), expresses a modest amount of E1A after the addition of 100 ng/ml of doxycycline (Dox), and this expression was sustained [Additional file 5: Supplemental Figure S4] after the cells had been subcultured three different times in preparation for the extraction of RNA and subsequent DNA microarray analysis (see below). After reaching a confluency of 40-60%, the cells in the absence of Dox were rendered quiescent by shifting them to medium containing 0.05% serum for a period of 60 hr, as previously described [4,5]. For DNA microarray analysis, quiescent cells in 0.05% medium for a period of 60 hr were treated with a 100 ng/ml of Dox for 8 hr and then harvested for the extraction of RNA.

### Isolation of RNA and Microarray Analysis

Total RNA was isolated by adding Trizol reagent (Invitrogen) directly to the monolayer of cells, and according to the manufacturer's instructions. The isolated RNA was then subjected to the RNeasy MinElute Cleanup Kit to ensure high-quality RNA, and afterwards, the RNA sample was subjected to a DNAse I treatment (DNAfree, Ambion) to remove all traces of contaminating DNA. The quantity and the quality of the RNA was assessed by NanoDrop 1000 (Thermo Scientific, Waltham, MA) and by the 18S/28S ribosomal peak intensity on an Agilent Bioanalyzer.

The microarray analysis was performed at the Cleveland Clinic Genomics core facility by using Agilent Mouse Whole Genome Arrays (Agilent, Santa Clara, CA) in a balanced block design. Total RNA (250 ng) was reverse transcribed into cRNA and biotin-UTP labeled using the Illumina Total Prep RNA Amplification kit (Ambion). cRNA was quantified using the NanoDrop 1000 spectrophotometer and its quality (size distribution) was further analyzed on a 1% agarose gel. Afterwards, the cRNA was hybridized to high-density oligonucleotide density arrays (an Illumina MouseRef8-v1.1 Expression BeadChip) containing 24, 613 probes or Target IDs, as well as ESTs sequences. The arrays were then washed, accordingly, and afterwards scanned using an Illumina BeadArray Reader.

### qRT-PCR Analysis

The genes showing p-values less than 0.05, and a change in gene expression of more than 2-fold when compared to control (determined by BeadStudio software) were selected for qRT-PCR analysis in order to validate the microarrays. The RNA used for qRT-PCR was isolated as described above for the microarray studies. cDNA was synthesized by using Invitrogen's SuperScript III First-Strand Synthesis kit with random hexamer primers. The resultant samples were then mixed with iQ™ SYBR^® ^Green Supermix (BioRad) and with specific primer sets purchased from Invitrogen and designed by programs, which included NCBI-BLAST, USCS Genome Browser, and Primer3. Primer pairs for the respective mRNAs under study were: Mcm3 (forward), 5'-TGACCTGCTCTTCATCATGC-3'; Mcm3 (reverse), 5'-CTGTG GCCCAGGATATC CACT-3'; Cdc6 (forward), 5'-AGGAGCCAGACAGTC CTCAA-3', Cdc6 (reverse), 5'-GGGTCAAAAGCAGCAAAGAG; Cyclin E (forward), 5'-CACAACATCCGACC CACAC-3', Cyclin E (reverse) 5'-GGCAG GTTGGTCATTCTGT-3'; Csf1 (forward), 5'-ACAACACCCCCAATGCTAAC-3', Csf1(reverse), 5'-ATGGAAAGTTCGGACAC AGG-3'; E2F1 (forward), 5'-GATCGAAGCTTTAATGGAGCG-3', E2F1 (reverse), 5'-CCCTTGCTTCAG AGAACAGG-3'; MCM7 (forward), 5'-CGGAGATCTA TGGACATGAA-3', MCM7(reverse),5'-CATAAGCAGATGTGGATGT-3'; GAPDH (forward), 5'-AAGGCCGAGAATGGGAAG-3', GAPDH (reverse), 5'-CTAAGCAGTT GGTGGTGCAG-3'; UNG (forward), 5'-TAATCAAGCTCACGGGCTCT-3', UNG (reverse), 5'-GCCAGGATGAACAAAACCAT-3'; PLK1 (forward), 5'-AATAGGG GATTTTGGCTTGG-3', PLK1(reverse), 5'-AATGGACCACACATCCACCT-3'; Skp2 (forward), 5'-GAAACGAGTCAAGGGCAAAG-3', Skp2 (reverse) 5'-AAGGAGCA GCTCATCTGGAA-3'. QRT-PCR analysis was performed by using an iCycler iQ™ machine (Bio-Rad), and relative changes were calculated by the ΔΔCt method using GAPDH or the 18S ribosomal RNA as a reference control gene.

### Statistical Analysis

#### Microarray Chips Pre-processing

Output files generated by the Illumina's BeadStudio application containing raw intensities were initially corrected for global normalization, variance-stabilization, and normality in order to identify and remove sources of systematic variation due to experimental artefacts (non-random or technical) and to subsequently perform proper statistical inferences. For this purpose, we used a Variance-Stabilizing Transformation (VST) which is a generalized-log(.) transformation (also known as arcsinh(.)) [30], ensuring that the variance is approximately *independent *of the mean intensity, followed by a Robust Spline Normalization (RSN) algorithm to the previously variance-stabilized data. The latter procedure is designed to combine the features of quantile and loess normalization. Details of the algorithm are published elsewhere [30]. The raw and preprocessed microarray datasets have been made MIAME-compliant and deposited in the Gene Expression Omnibus (GEO) under accession number GSE28420. These datasets can be accessed and downloaded at: http://www.ncbi.nlm.nih.gov/geo/query/acc.cgi?acc=GSE28420. To address the usual pre-processing issues upstream of the statistical analysis *per se*, we employed numerous implementations and algorithms available from the R project, which is a programming language and platform for statistical computing and visualization (http://www.r-project.org/), and from the Bioconductor project [31] (http://www.bioconductor.org/), which is a bioinformatics platform (also in the R language). Both platforms, which offer powerful statistics, bioinformatics, and visualization tools are freely available to academic users from the Comprehensive R Archive Network (CRAN) consortium (http://cran.r-project.org/). Specifically, for exploratory data analysis, pre-processing and quality control, we used the R package "Lumi" [32], which is especially designed to process the Illumina microarray data.

#### Pre-filtering and Bayesian Model Selection

The initial dataset contained 24,613 probes and ESTs uniquely identified by TargetIDs, and from which lists of pre-filtered probes satisfying certain (or a combination of) criterion were drawn: Before the Bayesian model selection analysis was used, a 'Present' or 'Absent' detection call was computed for each probe, similarly to the one described in [33]. The threshold for the 'Present' call *p*-value was set to *p *= 0.01. We selected those probes that have a 'Present' detection call (*p *= 0.01) in at least two out of 3 replicates and in at least one experimental group, leaving 10,632 probes. These probes were then used in a Bayesian model selection algorithm [15-17] to determine which of those were differentially expressed. The algorithm is currently implemented as stand-alone Java-based software freely available to academic users: Bayesian Analysis of Microarray (BAM) (http://www.bamarray.com). We report and use the *Group Mean Difference *and *Zcut *values in plots and tables. The *Group Mean Difference *value is simply calculated as the difference between the group means on the transformed scale (i.e. variance stabilization step as described in *pre-processing *section above). The *Zcut *value can be seen as Bayesian test statistic equal to the regular *Z*-test statistic from an ANOVA model for comparing two group means, and modulated by a real-valued coefficient bounded by 0 and 1, called a shrinkage factor. The closer the shrinkage factor is to 0, the more shrinkage there is, while the closer it is to 1, the closer *Zcut *is to the frequentist value [15-17].

#### Gene Annotations and Gene Ontologies (GO)

The Illumina microarray uses by default the TargetID identifiers for each 50mer sequence probe, which are not necessarily consistent among different versions of arrays. Therefore, we used the Nucleotide Universal Identifier (NuID) [34], which uniquely matches a 50mer oligonucleotide sequence and contains an error checking and self-identification code. By using the NuID, designers were able to build one annotation database for different versions of the human (or other species) chips [34]. Moreover, the NuID can be directly converted to the probe sequence, and used to get the most updated RefSeq matches and annotations. For functional annotations and functional analyses, we used the BIOCONDUCTOR platform (http://www.bioconductor.org/), which is a bioinformatics project (also in the R language). For the functional annotations and Gene Ontologies, we used the bioinformatics packages "annotate" and "GOstats". To perform enrichment analyses of GO categories among the specified gene symbol sets, we computed hypergeometric *p*-values for over or under-representation of each GO term in the specified category. The category enrichment was computed using a hypergeometric test statistic, based on the observed number of gene symbols representing that category and the expected number one would get by chance alone (i.e. under the null hypothesis of a random phenomenon).

### Pathways and Network Analysis

#### Canonical Pathways

Probe identifiers and *Zcut *values outputted from the Bayesian model of differentially regulated genes were up-loaded onto the Ingenuity Pathway Analysis (IPA version 8.5) website (http://www.ingenuity.com/ - Ingenuity Systems, Inc., Mountain View, CA) for canonical pathways and network analyses. Under the "General Settings" of the analysis, we first defined the "Reference Set" for our statistical tests (see *details *below). We used the set of 10,632 pre-filtered probes (see *filtering *section above) as a "User Dataset" since only those probes (genes) that were pre-selected had a chance of being monitored for enrichment and/or selected as differentially regulated (i.e. included in the test for differential expression). Therefore, this restricted set of genes only should be considered as "Reference Set" [35,36], which is to be distinguished from the total set of 24,613 uniquely identified probes and ESTs present in the MouseRef-8 v1.1, or from the Ingenuity Knowledge Database. For "Network Analysis", we included Direct and Indirect relationships as well as endogenous chemicals. The IPA "Data Source" was setup to "All knowledge databases", and to avoid analyzing noise in the dataset, we considered only those molecules/genes and/or relationships consistent with our experimental design, that is, "Species" was limited to "Mouse" and "Tissues/Cell Lines" to "Organ Systems" and "Uncategorized Cell Lines". The pathway analysis was performed on each individual set of induced and repressed molecules/genes separately to facilitate interpretations, as well as on the combined set of up/down-regulated molecules/genes to identify pathways in which a significant number of molecules/genes could be up- or down-regulated altogether. This left 385 up/down-regulated molecules/genes that were eligible for generating networks, and 1033 for functional and canonical pathways analysis. Moreover, of the down-regulated molecules/genes, 139 and 441 were eligible for generating networks and functional and canonical pathways, respectively, while 246 and 591 of the up-regulated molecules/genes were respectfully eligible for generating these categories as well. Significance of each individual pathway intrinsic to our list of differentially expressed genes was measured in IPA in two ways: (i) by a ratio (percentage) of those genes found in a given pathway of our list to all those constitutive of the corresponding canonical pathway; (ii) and by Fisher exact test 'right-tailed' *p*-value for the probability under the null hypothesis that there is no association between those set of genes found in a given pathway of our list with all those constitutive of the corresponding canonical pathway. The smaller the *p*-value, the less likely it is that the association is random and the more significant the association. The difference between the reported ratio and the *p*-values is a matter of percentage vs. probability. The ratio gives the amount of association; while the *p*-value gives a significance or confidence of association. Whenever a multiple-testing correction was required to assess significance of pathway enrichment (e.g. in a hypothesis generating query), we reported *adjusted p*-values using the Benjamini-Hochberg (BH) method [37].

#### Network Analysis

Differentially expressed genes were mapped in IPA to the global molecular network that was developed from information in the Ingenuity Knowledge Base. Networks for these genes were algorithmically generated based on their connectivity. A score equal to the negative log of the *p*-value of the right-tailed Fisher's exact test was assigned for each network. This score takes into account the number of eligible genes in our dataset and the size of the network to calculate the fit between each network and the genes in the dataset.

## Competing interests

The authors declare that they have no competing interests.

## Authors' contributions

J-ED, KZ, JS, and MLH conceived and designed the experiments. OY, LC, CN, MG, and JB performed the experiments. J-ED and MLH analyzed the data. J-ED and MLH wrote the paper. All authors have read and approved the final manuscript.

## Supplementary Material

Additional file 1 **Supplemental Table S1**. Entire list of significant genes differentially expressed in quiescent cells after E1A expression. Listing was generated by using the Bayesian ANOVA for microarrays (BAM). Transcripts are ranked by statistical significance (*Zcut *values) from top to bottom. Direction of change is indicated by the *Zcut *sign. Generalized-log-fold change values (glog(*FC*)) represent the corresponding *magnitude *of regulation of the selected genes. Click here for file

Additional file 2 **Supplemental Figure S1. Departure from normality of the distribution of differentially expressed genes**. This figure maps differentially expressed genes in quiescent cells after E1A induction onto a normal quantile-quantile plot. Genes found significantly up- and down-regulated by BAM analysis (2401 total, i.e. 1174 up-regulated and 1,227down-regulated) are highlighted in red or green, respectively, and the non-regulated genes are shown in grey. The top 50 regulated genes (up or down) from Table 1 in the text are named along with a few un-annotated genes (Rik identifiers). The solid blue line is the identity quantile line that passes through the first and third quartiles, showing departure from normality. Click here for file

Additional file 3 **Supplemental Figure S2. Validation of E1A-modulated genes identified by microarray analysis**. for validation, a set of nine genes (*CDC6, CCNE1, MCM3, E2F1*, *MCM7, SKP2, UNG, PLK1, and CSF1*) that were significantly regulated by E1A, as determined by BAM analysis, were tested by qRT-PCR. Left panel: volcano plot of absolute BAM Zcut values plotted versus log-fold-change-ratios on a log-equivalent transformed scale, denoted M = glog(S/Q), calculated either from microarray normalized-intensities (red) or qRT-PCR intensities (blue). Note that M values are on identical scale for each assay. Right panel: correlation plot of qRT-PCR fold-change versus microarray normalized-intensities fold-change. Dotted black lines represent the regression and identity lines. Click here for file

Additional file 4 **Supplemental Figure S3. The top metabolic functions affected by E1A in quiescent cells**. The stacked bar chart displays for each canonical pathway the number of genes that were found significantly up-regulated (red), and down-regulated (green) by Bayesian model selection. The molecules/genes in a given pathway that were not found in our list of significantly regulated genes are termed unchanged (grey) or not overlapping with our dataset (white). The numerical value at the top of each bar represents the total number of genes/molecules in the canonical pathway. The Benjamini-Hochberg (BH) method was used to adjust the right-tailed Fisher's exact *t*-test *p*-values, which measure how significant each pathway is. Click here for file

Additional file 5 **Supplemental Figure S4. Expression of E1A in E1A-inducible BALB/c 3T3 cells after treatment with Dox**. Nuclear extracts were prepared from E1A-inducible cells (Clone 13, passages 12, 18 and 31) after treatment with Dox (100 ng/ml) for 6 h. Extracts were then subjected to western blot analysis using M73, an antibody specific for E1A (4,5). The membrane was also probed with anti-GAPDH to monitor for equal loading of the extracts. Click here for file
