# Supplementary figures and images for: The dynamics of E1A in regulating networks and canonical pathways in quiescent cells
Source: BMC Res Notes. 2011 May 26;4:160. doi: 10.1186/1756-0500-4-160 (PMC3125344; doi:10.1186/1756-0500-4-160)

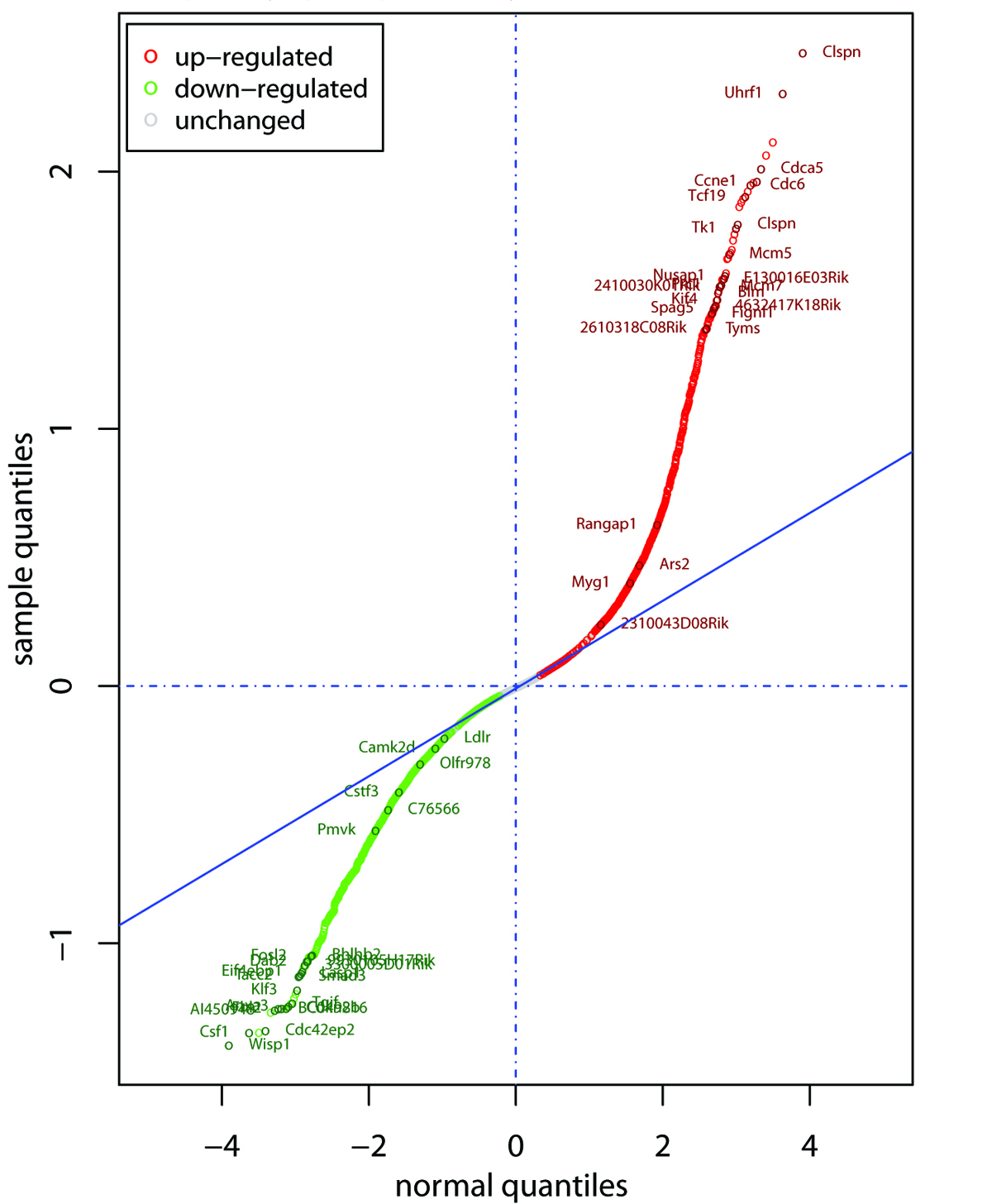

Supplement: Additional file 2 — Supplemental Figure S1. Departure from normality of the distribution of differentially expressed genes. This figure maps differentially expressed genes in quiescent cells after E1A induction onto a normal quantile-quantile plot. Genes found significantly up- and down-regulated by BAM analysis (2401 total, i.e. 1174 up-regulated and 1,227down-regulated) are highlighted in red or green, respectively, and the non-regulated genes are shown in grey. The top 50 regulated genes (up or down) from Table 1 in the text are named along with a few un-annotated genes (Rik identifiers). The solid blue line is the identity quantile line that passes through the first and third quartiles, showing departure from normality. [file 1756-0500-4-160-S2.JPEG]

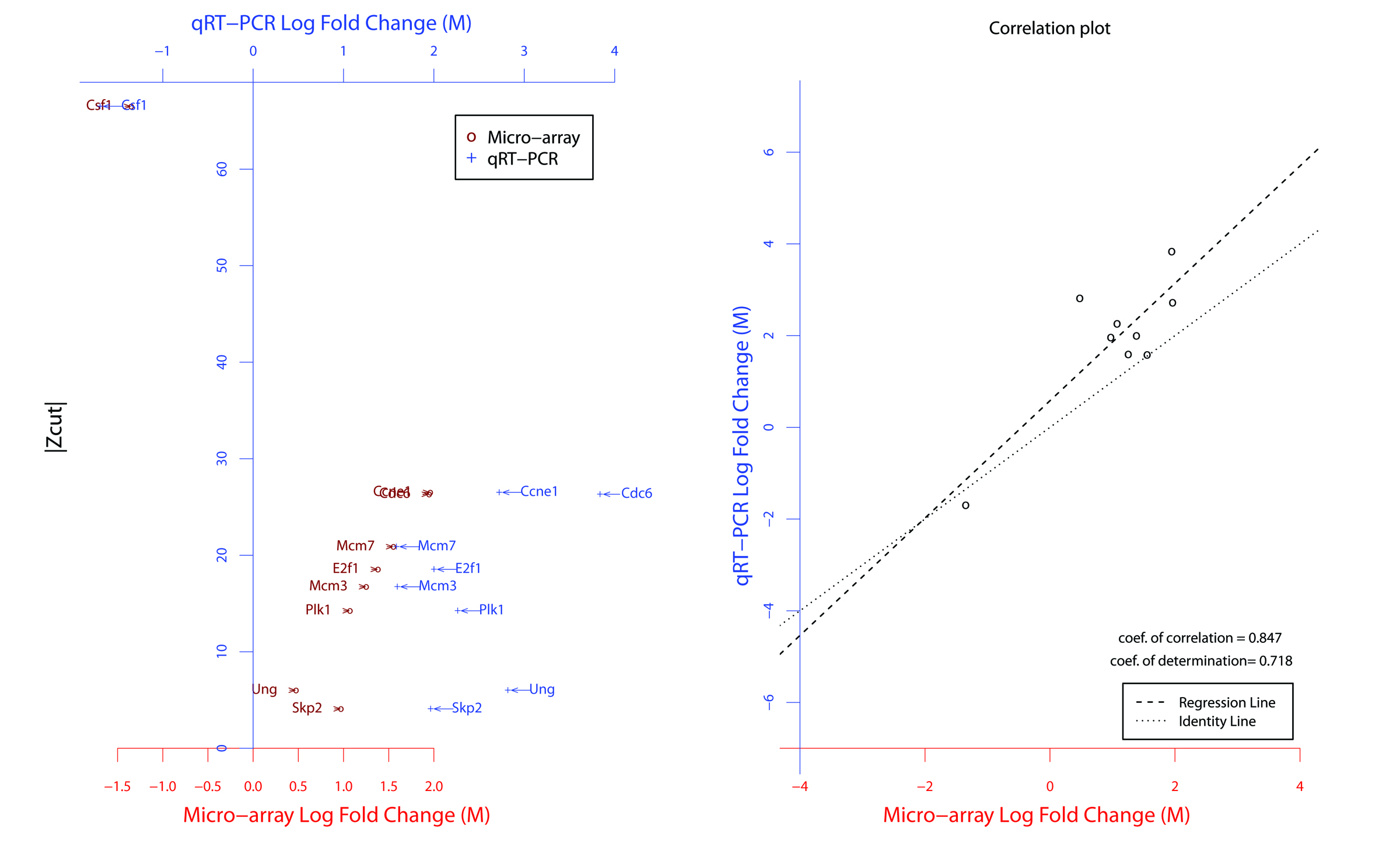

Supplement: Additional file 3 — Supplemental Figure S2. Validation of E1A-modulated genes identified by microarray analysis. for validation, a set of nine genes (CDC6, CCNE1, MCM3, E2F1, MCM7, SKP2, UNG, PLK1, and CSF1) that were significantly regulated by E1A, as determined by BAM analysis, were tested by qRT-PCR. Left panel: volcano plot of absolute BAM Zcut values plotted versus log-fold-change-ratios on a log-equivalent transformed scale, denoted M = glog(S/Q), calculated either from microarray normalized-intensities (red) or qRT-PCR intensities (blue). Note that M values are on identical scale for each assay. Right panel: correlation plot of qRT-PCR fold-change versus microarray normalized-intensities fold-change. Dotted black lines represent the regression and identity lines. [file 1756-0500-4-160-S3.JPEG]

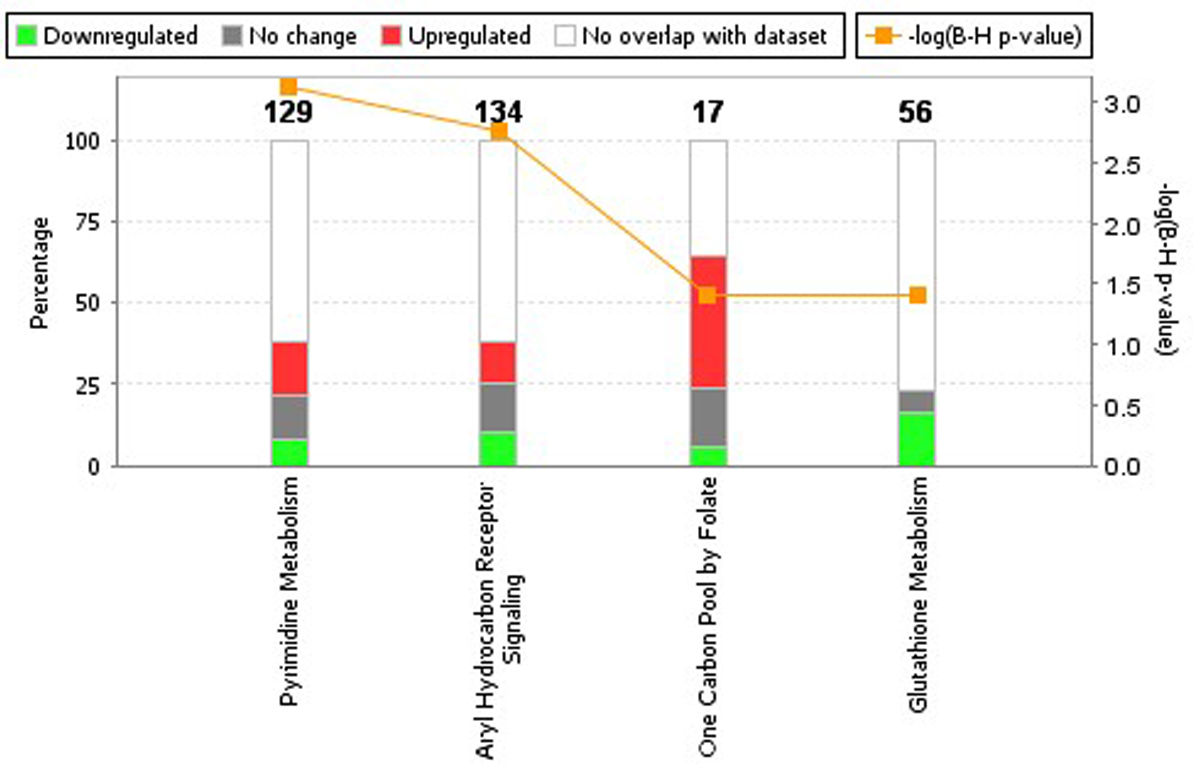

Supplement: Additional file 4 — Supplemental Figure S3. The top metabolic functions affected by E1A in quiescent cells. The stacked bar chart displays for each canonical pathway the number of genes that were found significantly up-regulated (red), and down-regulated (green) by Bayesian model selection. The molecules/genes in a given pathway that were not found in our list of significantly regulated genes are termed unchanged (grey) or not overlapping with our dataset (white). The numerical value at the top of each bar represents the total number of genes/molecules in the canonical pathway. The Benjamini-Hochberg (BH) method was used to adjust the right-tailed Fisher's exact t-test p-values, which measure how significant each pathway is. [file 1756-0500-4-160-S4.JPEG]

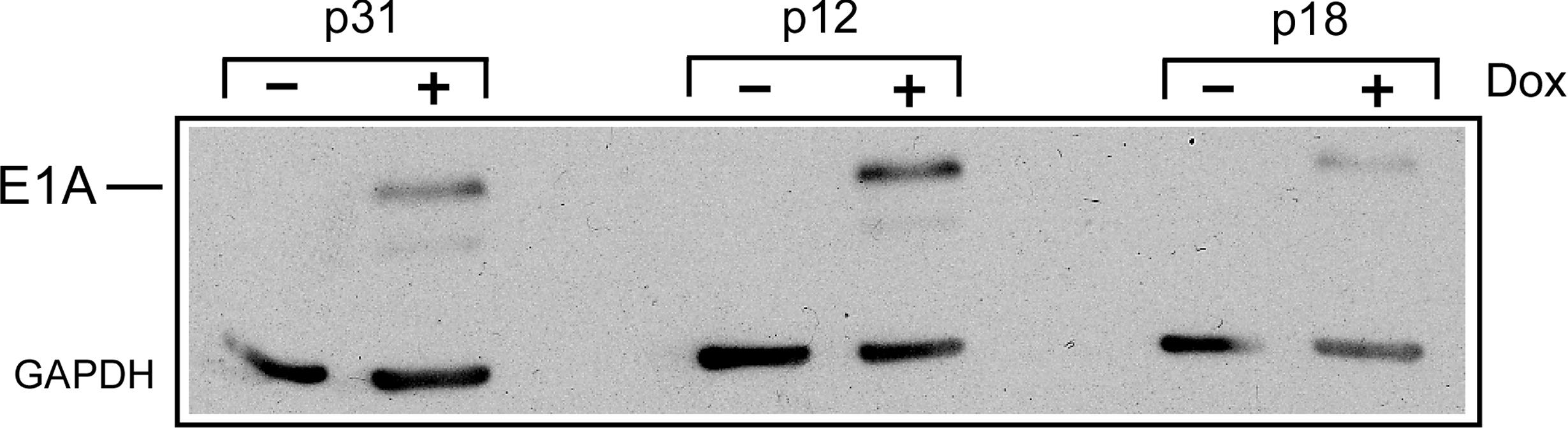

Supplement: Additional file 5 — Supplemental Figure S4. Expression of E1A in E1A-inducible BALB/c 3T3 cells after treatment with Dox. Nuclear extracts were prepared from E1A-inducible cells (Clone 13, passages 12, 18 and 31) after treatment with Dox (100 ng/ml) for 6 h. Extracts were then subjected to western blot analysis using M73, an antibody specific for E1A (4,5). The membrane was also probed with anti-GAPDH to monitor for equal loading of the extracts. [file 1756-0500-4-160-S5.JPEG]
